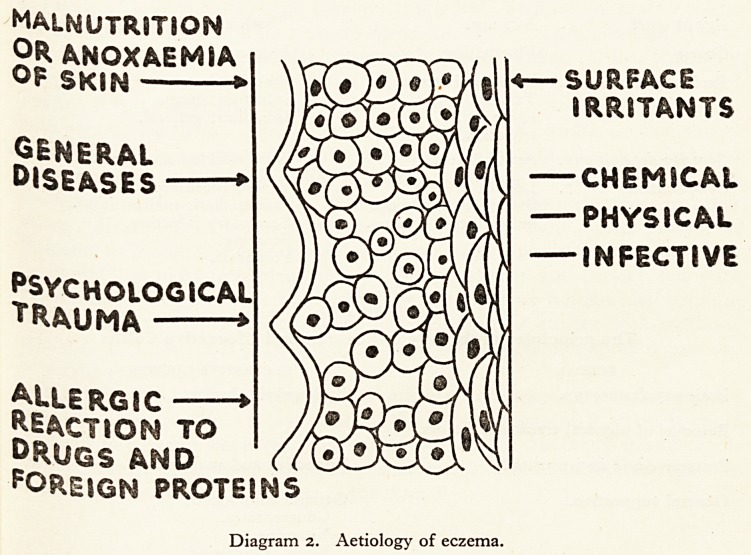# Eczema of the Colon: A New Conception of Ulcerative Colitis

**Published:** 1952-01

**Authors:** John Naish

**Affiliations:** Consultant Physician, Bristol Clinical Area


### ECZEMA OF THE COLON
### A New Conception of Ulcerative Colitis

BY

JOHN NAISH, M.D., M.R.C.P.
*Consultant Physician, Bristol Clinical Area*

The aetiology of chronic non-specific ulcerative colitis remains obscure. All attempts to discover the cause of the disease by direct pathological experiment and research have failed. Therefore it seems reasonable to broaden the inquiry and to seek for similarities between this and other disease processes.

Hitherto ulcerative colitis has been regarded as an infective condition in which the causative organism has eluded detection; its occurrence after bouts of bacillary dysentery, and the occasional acute onset of a fulminating form of the disease, support this view. But recently physicians and surgeons have turned their attention to the psychological aspects of the disease; for some time it has been known that these patients often have abnormalities of their personalities and reactions, and the question was debated whether these were the cause or the effect of the disease. Now, however, it seems well established that an abnormal psychological background is usually found in patients before they develop ulcerative colitis. More often than not, these patients are dependent, not aggressive, but querulous, and show a dull, prolonged resentment; they were model children, and frequently remain childish in adult life. (Paulley 1950, Mahoney *et al.* 1950.)

Furthermore there is often some emotional trauma immediately before the disease begins, although no satisfactory hypothesis has so far been advanced to explain the mechanism by which emotional trauma might lead to ulceration of the colon. There are other diseases in which emotional shock may lead to structural changes; acute gastric ulceration is one example of this sequence, and possibly other forms of peptic ulceration too. Certain forms of eczema may also develop as a reaction to emotional stress. If eczema is regarded as an abnormal form of surface reaction to a large variety of causes, it would be somewhat surprising if other membranes are not susceptible to similar changes. Perhaps the pathological processes in ulcerative colitis may be in certain aspects analogous to those found in eczema.

" Ulcerative " colitis may occur without ulceration. The existence of this verbal paradox is tacitly recognized but infrequently stressed by those familiar with the disease. Thus to quote Bockus (1944) " In the early stages of the disease one sees a wet, glistening, hyperaemic mucosa with oedema of both the mucosa and the submucosa resulting in varying degrees of apparent thickening ".

I have on many occasions observed the lower colonic mucosa of patients in the early stages of ulcerative colitis or of those enjoying a remission from the disease. In such cases no ulceration is to be seen and yet the mucosa is far from normal, for it is thickened, relatively immobile, wet, turgid and bleeds easily when stroked.

Eczema means *a boiling over* and this is just what appears to happen in ulcerative colitis too. The mucosa becomes deeply inflamed and oedematous, then it exudes, and finally ulcerates. The similarity between the pathological stages of eczema and of ulcerative colitis is shown in the Diagram I.

Diagram 1. Local pathology in eczema (*a*) and in ulcerative colitis (*b*).

The earliest change in both conditions appears to be an increase in the vascularity of the deeper layers, and exudation follows. In the skin this takes place through the bursting of minute vesicles; in the colon, exudation is at first through a granular mucosa which quickly breaks down to form an ulcerated surface. The colonic mucosa is naturally more delicate than the skin, and when it is oedematous and vascular it bleeds extremely easily, so that ulcers seem to form almost at a touch of the examining instrument. The skin with its tough waterproof integument is not subject to bleeding and ulceration unless secondary infection is severe.

It is also profitable to compare the known factors of aetiological importance in eczema and ulcerative colitis. The words eczema and dermatitis are here used as synonyms although the former is more often applied when constitutional factors predominate in the aetiology, and the latter when external traumatic factors are the most important.

Diagram 2. Aetiology of eczema.

It is clear that traumatic factors must be of much greater importance to the skin, the outer bodily covering, than they are to the lining of an internal tube such as the colon. On the other hand, ulceration, when it occurs, will lead to far greater evils in the colon than in the skin, because of the rich colonic bacterial flora and the vulnerability of the peritoneum. Hence there are a larger number of external causative factors in eczema, and there is greater frequency of complications in ulcerative colitis.

Complications of ulcerative colitis are far more severe than those of eczema. Dangerous bleeding, protein depletion, dehydration, perforation, stricture formation, localized ulcers, and polypoid and carcinomatous change, all give the disease a high mortality. Clearly the structure, contents and surroundings of the colon predispose to those destructive complications whereas the skin is essentially a defensive membrane immediately beneath which lie the less vulnerable parts of the body.

**Aetiology and Complications in Eczema and Ulcerative Colitis**

| | ECZEMA | ULCERATIVE COLITIS |
|---|---|---|
| *Family History* .. | In about 10 per cent of cases. | In about 2 per cent of cases. |
| *Personality* .. .. | Oversensitive.<br>Obsessive.<br>Active. | Oversensitive.<br>Neat; fastidious.<br>Obsessive.<br>Passive. |
| *Early History* .. | Commoner in only children.<br>History of broken home life common. | Commoner in only, or younger children.<br>Mother-fixation often present. |
| *Age at onset* .. | Any age. | Youth and middle age. |
| *Course* .. .. | Fluctuating. | Fluctuating. |
| *Exciting factors* .. | Emotional stress.<br>Trauma.<br>Ill-health.<br>Infection. | Emotional stress.<br>Infection; local.<br>Infection; general. |
| *Severity depends on* .. | Area affected. | Area affected and speed of onset. |
| *Complications* .. | Loss of protein.<br>Secondary infection.<br>Self-inflicted trauma. | Fever; Bleeding.<br>Protein loss; malnutrition.<br>Secondary infection.<br>Scarring.<br>Polyposis. |

THERAPY

**The principles of therapy in Eczema and Ulcerative Colitis**

| ECZEMA | ULCERATIVE COLITIS |
|---|---|
| Basic psychotherapy. | Basic psychotherapy. |
| Removal of physical traumatic factors. | |
| Protective and anti-pruritic. | Protective and analgesic. |
| General supportive. | General supportive:<br>Concentrates.<br>Vitamins.<br>Blood transfusion. |
| Chemotherapy for secondary infection. | Chemotherapy for secondary infection.<br>Surgical removal. |

There is a huge list of remedies which have been tried in ulcerative colitis. Usually the newest are at first successful, but later their popularity declines. This is a demonstration of the importance of the hopeful outlook and of the power of suggestion in treatment, a factor known to be true in asthma also. Eventual failure is the end of most remedies. The story of some of the most recent failures may be of interest, particularly the latest. *Thiouracil* was tried but had a short career, and extracts of dried intestines had scarcely more value than the two hundred or more other forms of tripe previously recommended for the disease. Yet another remedy has been tried and found wanting in recent years. It has been stated that *lyzozyme*, a mucolytic enzyme, is to be found in large quantities within the bowels of those suffering from ulcerative colitis.

(Meyer *et al.* 1948) (Gray *et al.* 1950), and that the quantities of lyzozyme within the bowel increased during emotional stress (Grace *et al.* 1949). Evidence was produced for this, and an hypothesis put forward—that lyzozyme in certain susceptible people was produced in excessive quantities as a response to emotional stress (Portis 1949). That being so, the lyzozyme would dissolve and break up the mucous secreted by the cells in the bowel wall. This mucous was considered to be the chief protection of the colonic mucosa against the digestive action of the succus entericus. The excessive production of lyzczyme would thus lead to enzymic digestion of the bowel wall. Experimentally such ulceration was produced in dogs by the application of lyzozyme to the colonic mucosa.

There are several flaws in this theory. If it is true, why does ulcerative colitis not affect the proximal rather than the distal colon? In fact, the disease in over fifty per cent of cases is confined to the left half of the colon. If it is true, why does the succus entericus not digest the small intestine as well as the large? Further it was known that lyzozyme was found in large quantities in association with granulation tissue. The colon in advanced ulcerative colitis is often almost entirely lined by granulation tissue. In spite of these difficulties, a search was made for substances which would inactivate lyzozyme. These were found in the kitchen with names such as *Dreft*, *Quix*, *Wisk*, *Fab*—detergents in other words. Treatment of a series of cases with one of these (Sod. Hexadecyl Sulphate is its scientific name), was begun; and as so often in the past the " new treatment " worked (Prudden 1950). Many patients got better; but later reports have not been hopeful and there seems to be no fundamental value in this treatment. Some patients have benefited from treatment with cortisone and A.C.T.H., but it is probable that this is yet another non-specific response to a new treatment.

What hope the future holds for the sufferer from ulcerative colitis, no one can tell. But until more is known about the cellular responses to stress, it would be right to regard ulcerative colitis as not primarily a disease of infection, invasion, trauma or degeneration, but as a disease of altered surface reaction—in most ways comparable to eczema of the skin.

### REFERENCES

Paulley, J. W. (1950): " Ulcerative colitis ", Gastro-enterology, **16**, 566.

Mahoney, V. P., Bockus, H. L., Ingram, Margaret, Hundley, J. W., and Yaskin, J. C. (1949): " A study of the personality in relation to ulcerative colitis ", *Gastro-enterology*, **13**, 547.

Grace, W. J., Seton, P. H., Wolf, Stewart, and Wolff, H. G. (1949): " Variations in concentration of lyzozyme with life situation and emotional state ", *Am. J. Med. Sc.*, **217**, 241.

Gray, S. J., Reifenstein, H. W., Connolly, E. P., Spiro, H. M., and Gordon Young, J. C. (1950): " Studies on lyzozyme in ulcerative colitis ", *Gastro-enterology*, **16**, 687.

Prudden, J. F., (1950): " The treatment of chronic ulcerative colitis with sodium hexadecyl sulfate ", *Gastro-enterology*, **15**, 426.

Portis, S. A. (1949): " Idopathic ulcerative colitis; newer concepts concerning its cause and management ", *J. Amer. Med. Ass.*, **139**, 208.

Meyer, K., Gellhorn, A., Prudden, J. F., Lehman, W. L., and Steinberg, A. (1948): " Lyzozyme activity in ulcerative alimentary disease ", *Amer. J. Med.*, **5**, 496.

## Figures and Tables

**Diagram 1. f1:**
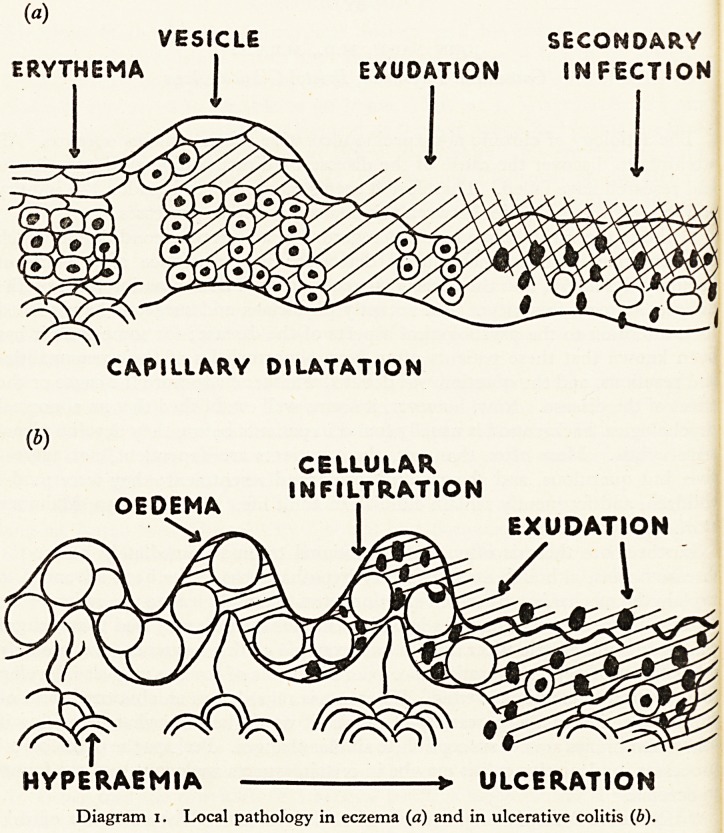


**Diagram 2. f2:**